# Replication-Competent ΔNS1 Influenza A Viruses Expressing Reporter Genes

**DOI:** 10.3390/v13040698

**Published:** 2021-04-17

**Authors:** Aitor Nogales, Michael Schotsaert, Raveen Rathnasinghe, Marta L. DeDiego, Adolfo García-Sastre, Luis Martinez-Sobrido

**Affiliations:** 1 Animal Health Research Centre (CISA), National Institute for Agriculture and Food Research and Technology (INIA), Valdeolmos, 28130 Madrid, Spain; 2 Department of Microbiology, Icahn School of Medicine at Mount Sinai, New York, NY 10029, USA; michael.schotsaert@mssm.edu (M.S.); rav.rathnasinghe@icahn.mssm.edu (R.R.); adolfo.garcia-sastre@mssm.edu (A.G.-S.); 3 Global Health and Emerging Pathogens Institute, Icahn School of Medicine at Mount Sinai, New York, NY 10029, USA; 4 Graduate School of Biomedical Sciences, Icahn School of Medicine at Mount Sinai, New York, NY 10029, USA; 5 Centro Nacional de Biotecnología, Consejo Superior de Investigaciones Científicas, 28049 Madrid, Spain; Marta.Lopez@cnb.csic.es; 6 Department of Medicine, Division of Infectious Diseases, Icahn School of Medicine at Mount Sinai, New York, NY 10029, USA; 7 The Tisch Cancer Institute, Icahn School of Medicine at Mount Sinai, New York, NY 10029, USA; 8 Texas Biomedical Research Institute, San Antonio, TX 78227, USA

**Keywords:** influenza A virus, NS1, reporter gene, luciferase, fluorescent protein, mCherry, interferon, STAT1

## Abstract

The influenza A virus (IAV) is able to infect multiple mammalian and avian species, and in humans IAV is responsible for annual seasonal epidemics and occasional pandemics of respiratory disease with significant health and economic impacts. Studying IAV involves laborious secondary methodologies to identify infected cells. Therefore, to circumvent this requirement, in recent years, multiple replication-competent infectious IAV expressing traceable reporter genes have been developed. These IAVs have been very useful for in vitro and/or in vivo studies of viral replication, identification of neutralizing antibodies or antivirals, and in studies to evaluate vaccine efficacy, among others. In this report, we describe, for the first time, the generation and characterization of two replication-competent influenza A/Puerto Rico/8/1934 H1N1 (PR8) viruses where the viral non-structural protein 1 (NS1) was substituted by the monomeric (m)Cherry fluorescent or the NanoLuc luciferase (Nluc) proteins. The ΔNS1 mCherry was able to replicate in cultured cells and in Signal Transducer and Activator of Transcription 1 (STAT1) deficient mice, although at a lower extent than a wild-type (WT) PR8 virus expressing the same mCherry fluorescent protein (WT mCherry). Notably, expression of either reporter gene (mCherry or Nluc) was detected in infected cells by fluorescent microscopy or luciferase plate readers, respectively. ΔNS1 IAV expressing reporter genes provide a novel approach to better understand the biology and pathogenesis of IAV, and represent an excellent tool to develop new therapeutic approaches against IAV infections.

## 1. Introduction

Influenza A viruses (IAVs) are enveloped viruses containing a segmented genome of eight single-stranded RNA molecules of negative polarity that belong to the *Orthomyxoviridae* family [1,2,3]. Currently, there are four circulating influenza virus types: A, B, C, and D (IAV, IBV, ICV, and IDV, respectively), which are able to infect multiple mammalian (IAV, IBV, ICV and IDV) and avian (IAV) species [4,5,6,7,8,9,10,11]. IAVs are classified into different subtypes based on the viral surface hemagglutinin (HA; 18 subtypes) and neuraminidase (NA; 11 subtypes) glycoproteins. All IAV subtypes (with the exception of H17N10 and H18N11 identified in fruit bats) have been isolated from wild aquatic birds, which are considered their natural reservoir [2,9,12,13]. In humans, IAV causes annual epidemics and occasional pandemics, representing a serious public health problem and associated economic impact [14,15,16,17,18,19]. Therefore, the implementation of new therapeutic approaches to prevent (vaccines) or control (antivirals) IAV infections as well as the development of novel biotechnological tools to study viral replication or pathogenesis are highly desirable [20,21,22,23,24,25,26,27,28,29,30].

Innate immune responses mediated by interferons (IFNs), IFN-stimulated genes (ISGs) and pro-inflammatory cytokines and chemokines are the first line of defense against viral infections, including IAV [23,31]. However, IAV encodes the multifunctional non-structural protein 1 (NS1) that is the main viral factor responsible for counteracting host innate immune responses induced during infection [32,33,34,35,36,37,38]. Consequently, viruses lacking NS1 or containing truncated forms of NS1 are affected in viral replication in most cells and hosts, except for those that are deficient in IFN production and/or signaling [21,25,27,38,39,40,41,42,43,44,45,46,47,48]. IAV NS1 is able to modulate cell innate immune responses through different mechanisms that can be host- and viral strain-dependent [11,16,32,34,36,49,50,51,52,53,54,55,56,57]. However, despite multiple studies with IAV NS1, there are many gaps related to its role in pathogenesis, replication or the ability to cross the host species barrier.

IAV NS1 protein is encoded from segment eight, or NS, as a linear transcript, which is also spliced to produce the nuclear export protein (NEP) [58,59,60,61,62]. The NS segment of multiple IAV strains has often been utilized for developing reporter-expressing viruses or vaccine candidates due to the knowledge accumulated about the expression strategy of the segment NS and the functions of its gene products, NS1 and NEP. Plasmid-based reverse genetics to engineer recombinant IAVs [7,27,63,64] have also had a significant impact on expanding our knowledge of the biology and pathogenesis of IAV, the identification of antivirals and the development of novel vaccine approaches. Moreover, these reverse-genetics strategies have been essential for the generation of replication-competent IAVs expressing one or two reporter genes, which have been used in multiple studies and have become a powerful approach to evaluate viral infections in vitro or in vivo, drug discovery, and vaccine efficacy [24,26,28,29,65,66,67,68,69,70,71]. To date, multiple strategies have been employed to develop recombinant replication-competent IAVs harboring fluorescent and/or bioluminescent reporter genes in different locations in the viral genome [29]. However, in all cases the recombinant constructs need to maintain intact the non-coding regions of viral genes, which are essential for viral replication and transcription. Moreover, packaging signals and other important sequences in the viral genome need to be preserved in order to recover infectious viruses [2,10,72,73,74,75].

Here, we describe and characterize, for the first time, the generation of a replication-competent NS1 deficient (ΔNS1) influenza A/Puerto Rico/08/1934 H1N1 (PR8) expressing a fluorescent (monomeric Cherry, mCherry) or bioluminescent (Nanoluc, Nluc) reporter gene. Viral infections with ΔNS1 mCherry and ΔNS1 Nluc were tracked in real-time using fluorescence microscopy or a luciferase plate reader, respectively. These replication-competent, reporter-expressing ΔNS1 IAVs were also evaluated for replication in cultured cells and for their ability to induce IFN responses, and in vivo using STAT1 deficient (STAT1^–/–^) mice. Our results demonstrate that these ΔNS1 reporter viruses represent an excellent option to directly study the biology of IAV, NS1 functions, and advance the discovery of countermeasures against IAV infections. Moreover, the flexibility of this system demonstrates the feasibility of generating recombinant IAVs expressing other foreign genes for their potential implementation as vaccine vectors for the treatment of other viral infections and, therefore, expanding the potential applications of ΔNS1 IAVs in vaccine development.

## 2. Materials and Methods

### 2.1. Cell Lines and Viruses

Human embryonic kidney 293T (293T; ATCC CRL-11268) and Madin-Darby canine kidney (MDCK, ATCC CCL-34) cells were grown in Dulbecco’s modified Eagle’s medium (DMEM; Mediatech, Inc. Manassas, VA, USA) supplemented with 5% fetal bovine serum (FBS; Atlanta Biologicals. Flowery Branch, GA, USA) and 1% penicillin (100 units/mL)–streptomycin (100 μg/mL)–2 mM l-glutamine (PSG; Mediatech, Inc.) at 37 °C in air enriched with 5% CO_2_. Influenza A/Puerto Rico/08/1934 (PR8) H1N1 expressing mCherry and ∆NS1 viruses were previously described [38,69]. Viral titers (plaque forming units, PFU, per milliliter) were determined by standard plaque assay in MDCK cells [11,24]. MDCK cells expressing the green fluorescent protein (GFP) and firefly luciferase (FFluc) proteins under the control of the IFNβ promoter (MDCK pIFNβ-GFP/IFNβ-FFluc) were previously described [44,47].

### 2.2. Construction of the NS Plasmids

To engineer a PR8 recombinant NS segment where the NS1 open reading frame (ORF) is removed and substituted by the reporter genes, we used our previously described pDZ-NS-2xBsmBI plasmid [69], which contains the NS1 ORF, without the stop codon or splice acceptor site, and two BsmBI sites followed by the porcine teschovirus-1 (PTV-1) 2A autoproteolytic cleavage site (ATNFSLLKQAGDVEENPGP) and NEP [69]. The mCherry or Nluc reporter genes were amplified by PCR using specific primers designed to introduce HindIII (located at the beginning of NS1) or BsmBI restriction sites. pDZ-NS-2xBsmBI, the PCR products were digested with BsmBI and HindIII and the reporter genes were cloned to generate the pDZ-∆NS1 mCherry or pDZ-∆NS1 Nluc plasmids for viral rescues. The final pDZ-∆NS1 mCherry or pDZ-∆NS1 Nluc constructs have the elements: 5′-non-coding region (NCR)/first 9 amino acids of NS1 (MDPNTVSSF)/reporter gene (mCherry or Nluc)/ PTV-1 2A (ATNFSLLKQAGDVEENPGP) /NEP/3’-NCR. Plasmid constructs were confirmed by sequencing (ACGT, Inc. Wheeling, IL, USA).

### 2.3. Rescue of Recombinant Reporter-Expressing ∆NS1 Viruses and Viral Infections

Recombinant ∆NS1 mCherry and Nluc PR8 viruses were rescued using previously described ambisense reverse genetics approaches [11,30,69]. Briefly, co-cultures (1:1) of 293T/MDCK cells (6-well plate format, 10^6^ cells/well, triplicates) were co-transfected, using LPF2000 (Invitrogen), in suspension with the eight PR8 ambisense pDZ-PB2, -PB1, -PA, -HA, -NP, -NA, -M, and -∆NS1 mCherry or Nluc plasmids. The recovered viruses were plaque purified on MDCK cells at 33 °C, and virus stocks were propagated in MDCK cells at 33 °C in a 5% CO_2_ atmosphere for 3 to 4 days. For viral infections, virus stocks were diluted in phosphate-buffered saline (PBS) supplemented with 0.3% bovine albumin (BA) and 1% penicillin-streptomycin (PS) (PBS-BA-PS). After viral infection, cells were maintained in DMEM supplemented with 0.3% BA, 1% PSG, and 1 μg/mL tosyl-sulfonyl phenylalanyl chloromethyl ketone (TPCK)-treated trypsin (Sigma, St. Louis, MO, USA) [11,30,69].

### 2.4. Virus Growth Kinetics

Multicycle virus growth kinetics were carried out in confluent MDCK cells (12-well plate format, 5 × 10^5^ cells/well, triplicates) infected with the indicated viruses at multiplicity of infection (MOI) of 0.001. After 1 h of virus adsorption at room temperature, cells were washed and overlaid with DMEM containing 0.3% BA and TPCK-treated trypsin and incubated at 33 °C or 37 °C. At the indicated times post infection (24, 48, 72, and 96 h), virus titers in the tissue culture supernatants were determined by immuno-focus assay (fluorescent focus-forming units, FFU/mL) using a mouse monoclonal antibody (MAb) against IAV NP (MAb HB-65; ATCC H16-L10-4R5) and a fluorescein isothiocyanate (FITC)-conjugated anti-mouse secondary antibody (Dako), as previously described [11,30,69]. The mean value and standard deviation (SD) were calculated using Microsoft Excel software. Cell monolayers were imaged to evaluate mCherry expression, and the presence of NLuc was quantified using Nano-Glo luciferase substrate (Promega, Madison, WI, USA) following the manufacturer’s specifications.

### 2.5. Protein Gel Electrophoresis and Western Blot Analysis

MDCK cells (12-well plate format, 5 × 10^5^ cells/well, triplicates) were mock-infected or infected (MOI of 2) with the indicated viruses and harvested at 18 h post infection (h p.i) to evaluate protein expression. Cells were lysed in passive lysis buffer (Promega) and frozen at −80 °C until use. Proteins from lysates were separated using 10% SDS-PAGE, transferred to a nitrocellulose membrane, blocked in 5% fat-free powdered milk dissolved in PBS containing 0.1% Tween-20 (PBS-T), and incubated overnight at 4 °C with the indicated primary MAb or polyclonal (PAb) antibodies against mCherry (rabbit PAb Raybiotech), NS1 (MAb 1A7) [56], NP (MAb HB-65; ATCC H16-L10-4R5) or actin (MAb A1978; Sigma). Bound primary antibodies were detected with anti-mouse or anti-rabbit horseradish peroxidase (HRP)-conjugated secondary antibodies (GE Healthcare, Madrid, Spain). Proteins were detected by chemo-luminescence (Thermo Fisher Scientific. Waltham, MA, USA) according to the manufacturer’s recommendations and photographed using a Kodak ImageStation digital imaging system.

### 2.6. Fluorescence and Indirect Immunofluorescence Assays

Confluent monolayers of MDCK cells (12-well plate format, 5 × 10^5^ cells/well, triplicates) were mock-infected or infected (MOI 2) with WT mCherry, ∆NS1 mCherry or ∆NS1 viruses. At 18 h.p.i, cells were fixed with 4% paraformaldehyde (PFA) and permeabilized with 0.5% Triton X-100 in PBS for 15 min at room temperature. After mCherry imaging, cells were blocked with 2.5% BA in PBS and stained using specific antibodies for NS1 (MAb 1A7) [56] or NP (MAb HB-65) and a FITC-conjugated rabbit anti-mouse polyclonal secondary antibody (Dako). 4′,6′-diamidino-2-phenylindole (DAPI, Research Organics) was used for nuclear staining. Images were captured using a fluorescence microscope (Nikon Eclipse TE2000), and processed using Adobe Photoshop CS4 (v11.0) software (San Jose, CA, USA).

### 2.7. Plaque Assays

Confluent monolayers of MDCK cells (6-well plate format, 10^6^ cells/well, triplicates) were infected with WT mCherry, ∆NS1 mCherry, ∆NS1 Nluc, or ∆NS1 viruses for 1 h at room temperature, overlaid with agar, and incubated at 33 °C. At 3 days p.i., cells were fixed overnight with 4% PFA and the overlays were removed. For visualization of mCherry, PBS was added and the plates were imaged with a ChemiDoc station (Bio-Rad, Madrid, Spain). To evaluate Nluc expression, cells were fixed and permeabilized (0.5% Triton X-100 in PBS) for 15 min at room temperature and prepared for immunostaining as previously described [24,26,69] using an NP MAb (HB-65), and after extensive wash with PBS, with an NLuc pAb (kindly provided by Promega). Inmuno-staining was developed using vector kits (Vectastain ABC kit for mouse or rabbit antibodies and DAB HRP substrate kit; Vector) following the manufacturers’ specifications.

### 2.8. Cell-Based IFN Bioassay

To evaluate the induction of IFNβ in vitro, MDCK cells (12-well plate format, 5 × 10^5^ cells, triplicates) expressing GFP and FFluc reporter genes under the control of the IFNβ promoter (MDCK IFNβ-GFP/IFNβ-FFluc) [44,47] were mock-infected or infected (MOI 2) with WT or ∆NS1 mCherry viruses for 12 h. Activation of the IFNβ promoter was determined by FFluc expression in cell lysates using a Promega luciferase reporter assay and a microplate reader (DTX880; Beckman Coulter. Brea, CA, USA). mCherry (from virus) and GFP (from cells) expression were evaluated using a fluorescence microscope as described above. Mean values and SDs were calculated using Microsoft Excel software (Redmond, WA, USA). IFNβ promoter activation was represented as fold change relative to mock-infected cells.

### 2.9. Mouse Studies

STAT1^−/−^ C57BL/6 mice were bred locally at The Icahn School of Medicine at Mount Sinai. Mice were housed in a barrier facility under specific pathogen free conditions. All mouse procedures were approved by the Icahn School of Medicine at Mount Sinai Institutional Animal Care and Use Committee (IACUC) and performed in accordance with the IACUC guidelines. Ten-week-old STAT1^−/−^ C57BL/6 mice were anesthetized by intraperitoneal (IP) administration of ketamine, xylazine diluted with PBS (1:1:8) before intranasal (IN) inoculation of 10^4^ PFU of the indicated viruses. Mice (*n* = 5/group) were examined daily for morbidity (body weight loss) and mortality for 4 days. Mice showing more than 25% body weight loss were euthanized humanely. To evaluate viral replication, mice (*n* = 3/group) were euthanized by IP administration of Pentabarbitol. The lungs were excised at days 1, 2 or 4 p.i., and viral titers were determined by plaque assay in MDCK cells as previously described.

## 3. Results

### 3.1. Generation of a Recombinant ΔNS1 PR8 Virus Expressing mCherry

**Figure 1:**
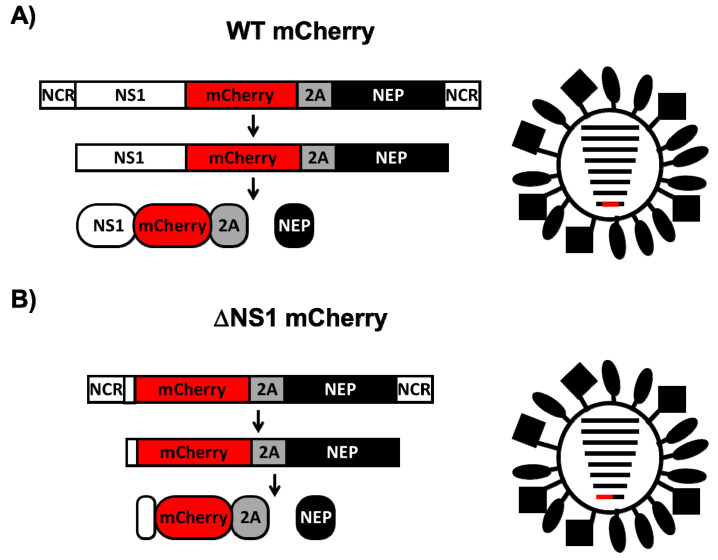
Schematic representation of the influenza PR8 WT (**A**) and ∆NS1 (**B**) mCherry segments (left) and viruses (right). The viral NS1 and NEP are indicated with white and black boxes, respectively. Sequences of mCherry and PTV-1 2A are indicated in red and gray boxes, respectively. NCR: non-coding regions.

In a previous work, we described the generation and characterization of a replication-competent mCherry fluorescent-expressing PR8 virus, where the NS1 protein was fused to mCherry (Figure 1A) [69]. Because the NS segment, which encodes NS1 and NEP, is alternatively spliced to produce NEP, the porcine teschovirus-1 (PTV-1) 2A autoproteolytic cleavage site was inserted between NS1 and NEP so that both proteins (NS1 and NEP) are transcribed from the same mRNA and translated individually (Figure 1A) [69]. Then, mCherry was cloned as a fusion to the C-terminus of NS1 to generate a recombinant PR8 WT virus expressing mCherry (Figure 1A). This strategy allows for collinear expression of NS1-mCherry and NEP from the same viral transcript.

In order to engineer a replication-competent ΔNS1 PR8 virus expressing mCherry, the viral NS segment was modified to replace the NS1 by mCherry without the stop codon (Figure 1B). Then, the PTV-1 2A sequence was inserted between the reporter gene and NEP allowing the expression of both proteins [69] (Figure 1B). For the successful recovery of replication-competent reporter-expressing ΔNS1 mCherry virus, we did not disrupt the NCR at the 5′ end of the viral segment, and the first nine amino acids of NS1 were maintained in the novel recombinant ΔNS1 NS segment (Figure 1B). We then used PR8 plasmid-based reverse genetics to generate a recombinant NS1-deficient virus expressing mCherry (ΔNS1 mCherry).

### 3.2. In Vitro Characterization of ΔNS1 mCherry

**Figure 2:**
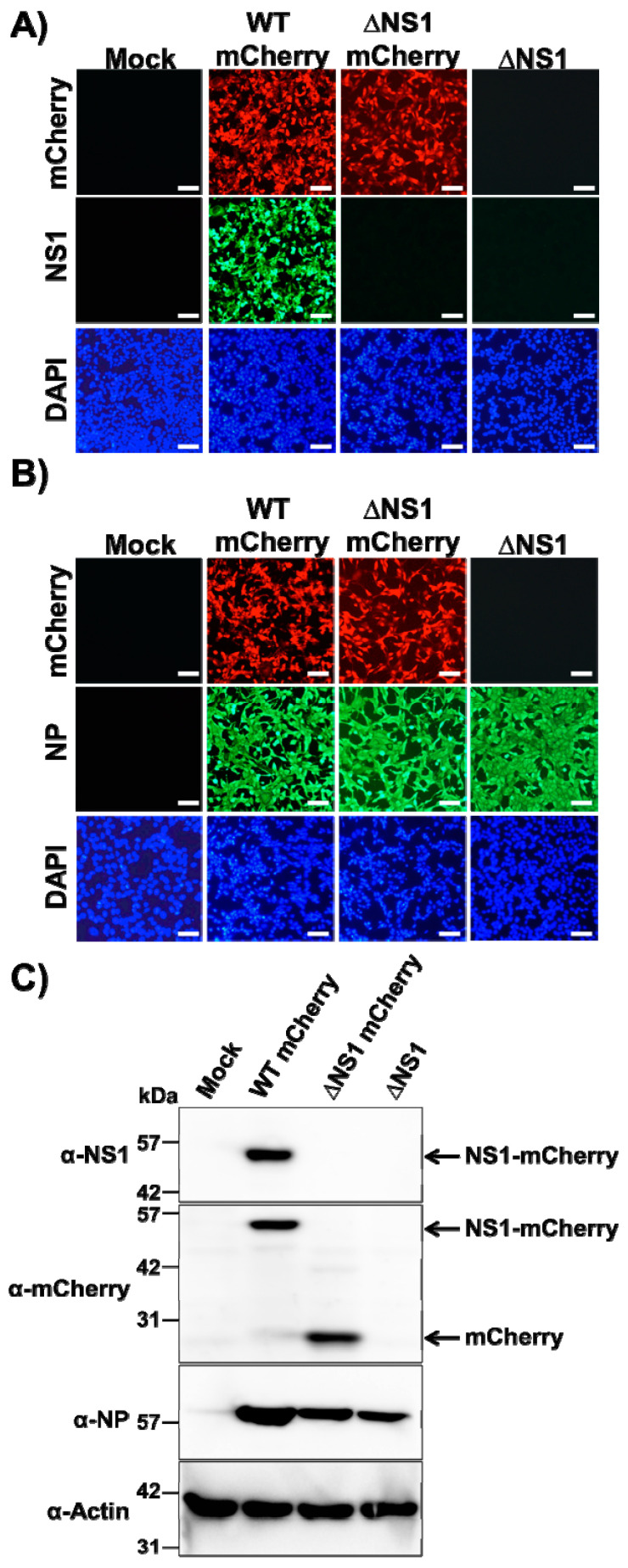
In vitro characterization of ∆NS1 mCherry: (**A**,**B**) Analysis of protein expression by fluorescence and immunofluorescence: MDCK cells (12-well plate format, 5 × 10^5^ cells/well, triplicates) were non-infected (Mock) or infected (MOI 2) with WT mCherry, ∆NS1 mCherry or ∆NS1 viruses. At 18 h p.i., cells were fixed and permeabilized and visualized for mCherry expression. After mCherry imaging, cells were stained with NS1 (**A**) or NP (**B**) MAbs. DAPI was used for nuclear staining. Representative images (20× magnification) are included. Scale bar, 50 µm. (**C**) Analysis of protein expression by Western blot: MDCK cells (12-well plate format, 5 × 10^5^ cells/well, triplicates) were mock-infected or infected (MOI 2) as above and protein expression levels of NS1, NP and mCherry were evaluated using protein specific antibodies. Cellular actin was used as a loading control. Numbers indicate the size of molecular markers in kDa. Arrows indicates the presence of NS1-mCherry (WT mCherry) and mCherry (∆NS1 mCherry).

We next evaluated if ΔNS1 mCherry could be directly visualized in infected cells to track viral infection (Figure 2). To that end, confluent monolayers of MDCK cells were mock-infected or infected (MOI 2) with WT or ΔNS1 mCherry viruses and, at 18 h p.i, cells were analyzed by indirect immunofluorescence using specific antibodies for IAV NS1 (Figure 2A) or NP (Figure 2B), or direct fluorescence mCherry expression. As expected, NS1 expression was detected only in cells infected with WT mCherry virus (Figure 2A). Moreover, cells infected with WT and ΔNS1 mCherry viruses were fluorescent upon direct examination under a fluorescent microscope (Figure 2A,B). Importantly, the subcellular localization of the viral NP was similar for cells infected with both WT and ΔNS1 mCherry viruses (Figure 2B). As expected, cells infected with ΔNS1 did not express mCherry or the viral NS1 but were stained with the NP MAb. Mock-infected cells were negative when stained with the IAV NS1 or NP MAbs. The identity of ΔNS1 mCherry was then confirmed by Western blot (Figure 2C). Cell extracts from MDCK cells mock-infected or infected with WT mCherry, ΔNS1 mCherry or ΔNS1 viruses were examined at 18 h p.i. using antibodies specific for NS1 or mCherry. Antibodies against the viral NP or cellular actin were used as infection and loading controls, respectively. A specific band corresponding to NS1-mCherry fusion protein was observed with the anti-NS1 antibody only in WT mCherry infected cells. Western blot examination with the anti-mCherry antibody detected specific bands in cell extracts from WT or ΔNS1 mCherry viruses, and the molecular size of those bands corresponded with the expected size for NS1-mCherry or mCherry proteins, respectively. As expected, in cell extracts from MDCK cells infected with ΔNS1 virus, we did not detect the expression of either NS1 or mCherry. Viral NP was clearly detected in cells infected with the three viruses. Altogether, these data demonstrate that a replication-competent ΔNS1 virus expressing mCherry can be recovered and that MDCK cells infected with ΔNS1 mCherry express high levels of mCherry.

**Figure 3:**
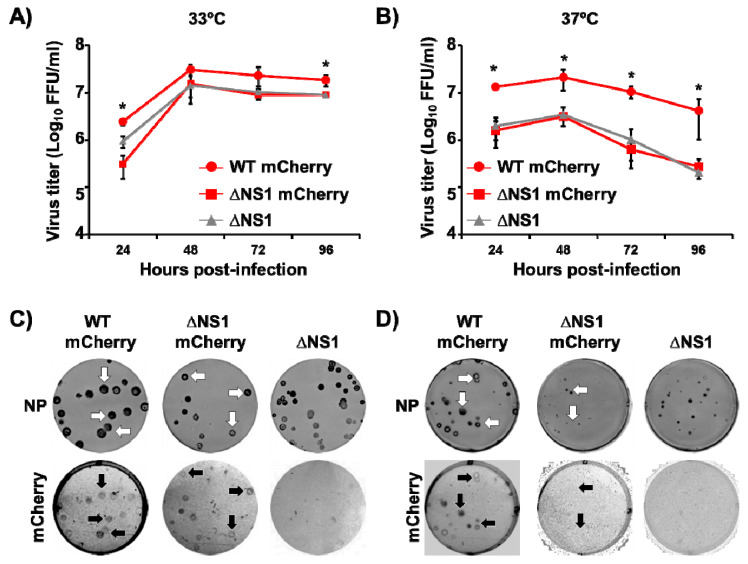
Growth kinetics and plaque phenotype of ∆NS1 mCherry. (**A**,**B**) Multicycle growth kinetics: MDCK cells (12-well plate format, 5 × 10^5^ cells/well, triplicates) were infected (MOI 0.001) with WT mCherry, ∆NS1 mCherry or ∆NS1 viruses. Virus titers in tissue culture supernatants from infected cells at 33 °C (**A**) or 37 °C (**B**) at the indicated times p.i. were calculated by immuno-focus assay (FFU/mL). * *p* < 0.05 (WT mCherry versus ΔNS1 mCherry or ΔNS1) using Student’s *t* test (*n* = 3 per time point) from Microsoft Excel. (**C**,**D**) Plaque phenotype: MDCK cells (6-well plate format, 1 × 10^6^ cells/well, triplicates) were infected with ~25 FFU of WT mCherry, ∆NS1 mCherry or ∆NS1 viruses and incubated at 33 °C (**C**) or 37 °C (**D**) for 3 days. Plaques were evaluated by immunostaining using a MAb against IAV NP (MAb HB-65) or by fluorescence mCherry expression. For WT mCherry or ∆NS1 mCherry viral infections, arrows indicate correlation between NP positive (top, white arrows) and mCherry fluorescent (bottom, black arrows) plaques.

To evaluate the replication properties of ΔNS1 mCherry virus, multicycle growth kinetics were performed at two different temperatures (33 °C and 37 °C) (Figure 3A,B, respectively). To that end, confluent monolayers of MDCK cells were infected (MOI 0.001) with WT mCherry, ΔNS1 mCherry or ΔNS1 PR8 viruses and presence of virus in tissue culture supernatants were determined at different h p.i. All recombinant PR8 viruses reached high and similar titers in MDCK cells at 33 °C, although WT mCherry showed statistically significant higher viral titers at 24 and 96 h p.i (Figure 3A). Therefore, the introduction of the mCherry gene did not significantly affect in vitro ΔNS1 mCherry growth kinetics and viral replication in MDCK cells at 33 °C (Figure 3A). However, at higher temperatures (37 °C), WT mCherry virus replicated at significant higher titers than ΔNS1 mCherry or ΔNS1 viruses (Figure 3B). This was expected, since previous studies have shown that NS1 deficient, truncated or mutant viruses are temperature sensitive [39,76,77,78,79]. When we evaluated the plaque phenotype of WT mCherry, ΔNS1 mCherry and ΔNS1 PR8 viruses in MDCK cells, the plaque sized of all viruses were similar at 33 °C (Figure 3C). Notably all plaques detected using the NP MAb expressed mCherry in both the WT and the ΔNS1 mCherry viruses (Figure 3C, white and black arrows), indicating that all infectious viruses express mCherry. Conversely, the plaque sizes of WT mCherry virus at 37 °C were bigger than those of the ΔNS1 mCherry and ΔNS1 viruses. As expected, the plaque phenotype of the ΔNS1 mCherry and ΔNS1 viruses at 37 °C was smaller than those at 33 °C [39,76,77,78,79]. As expected, mCherry expression from ΔNS1 mCherry virus was barely detected (Figure 3D).

### 3.3. Ability of ΔNS1 mCherry to Inhibit IFNβ Promoter Activation

**Figure 4:**
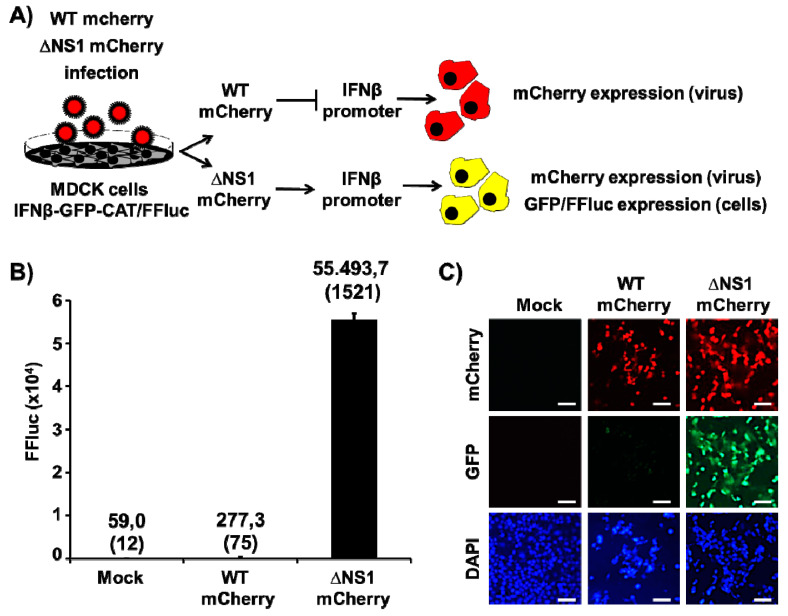
Analysis of IFNβ promoter activation by ∆NS1 mCherry. (**A**) Schematic representation of the IFNβ bioassay. MDCK pIFNβ-GFP/IFNβ-FFluc cells (12-well plates, 5 × 10^5^ cells/well, triplicates) were mock-infected or infected (MOI 2) with WT or ∆NS1 mCherry viruses and analyzed for IFNβ promoter activation at 12 h p.i. (**B**) IFNβ promoter activation (FFluc expression): Cell extracts from mock and virus infected-cells were evaluated for IFNβ promoter activation by FFluc expression. Data show means and (SD) of the results from triplicate samples. (**C**) IFNβ promoter activation (GFP expression): Activation of IFNβ promoter (GFP) and infection with WT or ∆NS1 viruses (mCherry) was visualized using a fluorescence microscope. Mock infected cells were included as internal control. Representative fields (20× magnification) are shown. Scale bar, 100 µm.

Since the primary role of IAV NS1 protein is to inhibit IFN and host antiviral responses during viral infection [11,32,37,38], we explored the effect of substituting NS1 with mCherry, on the capacity of the virus to inhibit induction of IFNβ by using a well-characterized cell-based IFNβ bioassay (Figure 4A). For this, MDCK IFNβ-GFP/IFNβ-FFluc cells, which express FFluc and GFP reporter genes under the control of the IFNβ promoter, were mock-infected or infected (MOI 2) with WT or ΔNS1 mCherry PR8 viruses and, at 12 h p.i., IFNβ promoter activation was evaluated by examining FFluc (Figure 4B) or GFP (Figure 4C) expression. Unsurprisingly, FFluc and GFP expression were higher in cells infected with ΔNS1 mCherry than in cells infected with WT mCherry (Figure 4B,C, respectively), where viral NS1 was able to inhibit IFNβ promoter activation [11,32,37,38,69], indicating that ΔNS1 mCherry was not able to control IFNβ promoter activation during viral infection. Infection of MDCK cells with WT mCherry or ΔNS1 mCherry viruses was confirmed by mCherry expression (Figure 4C).

### 3.4. ΔNS1 mCherry Infection in Mice

**Figure 5:**
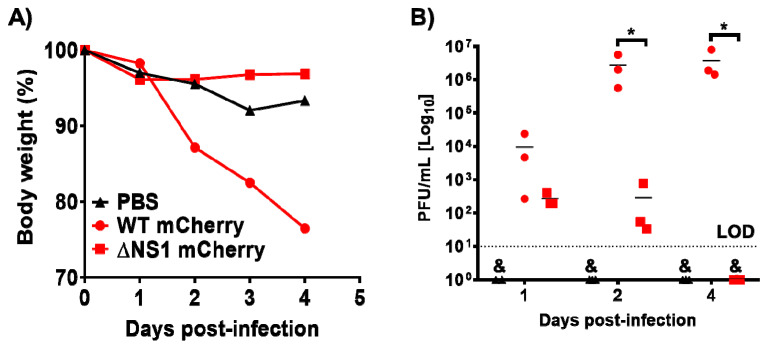
Virulence and replication of ΔNS1 mCherry in STAT1^−/−^ C57BL/6 mice. (**A**) Virulence: Groups of STAT1^−/−^ C57BL/6 mice (*n* = 5) were mock-infected (PBS) or infected IN with 10^4^ PFU of WT or ΔNS1 mCherry viruses, and body weight loss was evaluated for four days. Mice that lost 25% or greater of their initial body weight were sacrificed. (**B**) Viral replication: Groups of STAT1^−/−^ C57BL/6 mice (*n* = 3) were similarly infected as those described in A, sacrificed at days 1, 2 and 4 p.i. and lungs were harvested, homogenized and used to quantify viral titers (PFU/mL). Symbols represent data from individual mice. Bars represent the means of lung viral titers. & Infectious virus was not detected. Black dotted lines indicate the limit of detection, LOD (100 PFU/mL). * *p* ≤ 0.05 using Student’s *t*-test.

Signal transducer and activator of transcription 1 (STAT1) is a transcription factor that plays a key role in IFN signaling, therefore, cells lacking STAT1 respond aberrantly to IFN [32,38,80,81]. It has been shown that STAT1^−/−^ C57BL/6 mice fail to mount efficient responses to IFN and they are susceptible to viral infections [32,38,80,81]. Moreover, it has been reported that ΔNS1 IAVs are not virulent in WT C57BL/6 mice [11], while they are able to replicate and cause morbidity and mortality in STAT1^−/−^ C57BL/6 mice [38]. In addition, IAV WT viruses are able to replicate at a higher extent and cause greater morbidity and mortality in STAT1^−/−^ C57BL/6 mice [81,82]. Thus, we evaluated if ΔNS1 mCherry was pathogenic and able to replicate in STAT1^−/−^ C57BL/6 mice (Figure 5). To that end, animals were infected IN with 10^4^ PFU of WT or ΔNS1 mCherry viruses, and body weight loss was monitored daily for four days (Figure 5A). All mice inoculated with WT mCherry rapidly lost weight and succumbed to viral infection, whereas animals inoculated with ΔNS1 mCherry did not significantly loss weight and all of them survived viral infection (Figure 5A). To evaluate whether the observed attenuation correlated with virus replication, we also evaluated viral titers in the lungs of infected STAT1^−/−^ C57BL/6 mice (Figure 5B). To that end, groups of mice (*n* = 3) were inoculated IN with 10^4^ PFU of WT or ΔNS1 mCherry viruses, and viral titers were evaluated at 1, 2, and 4 days p.i. Animals infected with the WT mCherry showed significant higher viral titers at 2 and 4 days p.i., compared to mice infected with ΔNS1 mCherry. These data indicate that ΔNS1 mCherry is able to replicate in STAT1^−/−^ C57BL/6 mice, but at a lower extent than WT mCherry.

### 3.5. Generation and Characterization of a Replication-Competent ΔNS1 Nluc

**Figure 6:**
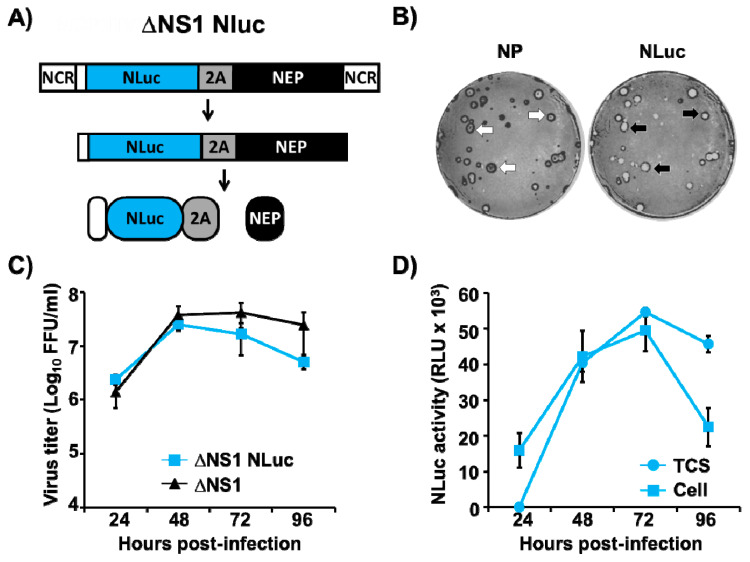
A NLuc-expressing ∆NS1 virus (∆NS1 NLuc). (**A**) Schematic representation of ∆NS1 NLuc: Schematic representation of ∆NS1 NLuc where NS1 was substituted with Nluc in the NS segment, using the same approach as that used to generate the ΔNS1 mCherry (Figure 1). The viral NEP is indicated with a black box. Sequences of PTV-1 2A are indicated with a gray box. NCR: non-coding regions. (**B**) Plaque assays: NP (left) and Nluc (right) expression in MDCK cells infected with ∆NS1 NLuc were evaluated at 3 days p.i. by immunostaining using a MAb against IAV NP or a pAb against Nluc. Arrows indicate correlation between NP (white arrows) and Nluc positive (black arrows) plaques. (**C**) Multicycle growth kinetics: Virus titers from tissue culture supernatants of MDCK cells (12 well plates, 5 × 10^5^ cells/well, triplicates) infected (MOI 0.001) with ∆NS1 Nluc or ∆NS1. Viral replication was quantified at the indicated times p.i. by immuno-focus assay (FFU/mL). Data represent the means ± SD of triplicates. (**D**) Quantification of NLuc expression: Nluc activity in the tissue culture supernatants (TCS) and cell lysates (Cell) of MDCK cells (12 well-plate format, 5 × 10^5^ cells/well, triplicates) infected (MOI of 0.001) with ∆NS1 NLuc were quantified at the indicated times p.i. Data represent the means ± SD of triplicates.

Although multiple reporter genes with different characteristics are used to generate replication-competent IAVs, fluorescent or bioluminescent reporters are usually the favorite options [24,26,28,29,68,71]. Given that, for quantitative purposes, luciferases are usually more convenient than fluorescent reporters [24,83,84,85,86], and to validate the use of this novel technology for the generation of other reporter-expressing ΔNS1 viruses, we attempted the generation of a ΔNS1 expressing Nluc [87] using the same experimental approach we used to generate ΔNS1 mCherry (Figure 6A). A ΔNS1 Nluc was successfully recovered (Figure 6B), and in MDCK cells, ΔNS1 Nluc showed similar growth kinetics than ΔNS1 virus at 33 °C (Figure 6C). Importantly, MDCK cells infected with ΔNS1 Nluc virus expressed high levels of Nluc, which was detected either in the tissue culture supernatants or in cell extracts (Figure 6D). These data demonstrate the feasibility of our system to engineer ΔNS1 viruses expressing different reporter genes.

## 4. Discussion

Host innate immune defenses were activated upon infection limit virus replication and infection [32,88]. However, IAV has evolved to encode proteins such as PA-X, PB1-F2 or, most importantly, NS1 to counteract the antiviral countermeasures of the targeted host during infection [32]. IAV NS1 is a multifunctional protein highly expressed in infected cells, whose major role is to prevent the induction of IFN and the activities of ISG proteins [11,32,36,37], representing an important viral pathogenesis factor [11,34,50,51,52,53]. IAVs, where partial or the entire NS1 gene was removed, have been widely used as potential vaccines [21,25,39,43,45,46,47,48]. These recombinant IAVs have also allowed researchers to evaluate specific functions and mechanisms of action for NS1 from different IAV strains. Interestingly, in a recent report, we were able to recover a recombinant IAV expressing IAV, IBV, ICV, and IDV NS1 proteins, and show that recombinant IAVs expressing heterotypic (IBV, ICV, and IDV) NS1 proteins were highly attenuated in a mouse model of infection [11]. All these technological advances and studies highlight the importance of increasing our knowledge about IAV genome flexibility to incorporate foreign sequences, including reporter genes, and identify new viral protein functions, as well as the development of novel tools to evaluate viral infection and pathogenesis [24,26,28,29,65,66,68,69,70,83,84,85].

Studying IAV requires the use of time-consuming methodologies to identify virus-infected cells, and usually researchers obtain only a limited snapshot of the viral infection process. To circumvent this requirement and track viral dynamics in vitro or in vivo, replication-competent IAVs expressing easily traceable fluorescent or luciferase reporter genes have been engineered and amply used in the field to study IAV infection or pathogenesis, the identification of antivirals and neutralizing antibodies, or the development of novel vaccines [24,26,28,29,65,66,68,69,70,83,84,85]. However, the majority of these recombinant viruses were based in WT viruses, where the reporter gene is expressed from one viral segment independently or fused to a viral protein.

Here, a replication-competent ΔNS1 IAV expressing mCherry fluorescent protein (Figure 1) was generated and characterized in vitro and in vivo. Our studies indicate that although the replication of ΔNS1 mCherry was not severely impaired as compared to WT mCherry in MDCK cells at 33 °C, viral replication was reduced at 37 °C (Figure 3), as has been previously described for other ΔNS1 IAVs [39]. Moreover, ΔNS1 mCherry was not able to inhibit IFNβ promoter activation to levels comparable to WT mCherry (Figure 4). In addition, our results in vivo showed that virulence of ΔNS1 mCherry was also reduced in STAT1^−/−^ C57BL/6 mice as compared with animals infected with WT mCherry (Figure 5), where ΔNS1 mCherry was cleared four days after infection, while high levels of replication for WT mCherry were observed. Notably, these in vivo data suggest that reporter-expressing IAV could be used to easily identify cell types more permissive to being infected by ΔNS1 viruses, and therefore with weaker innate immune responses than other types of cell.

The selection of one specific reporter gene depends on the type of study and several variables are usually taken in consideration, including the application for in vitro or in vivo studies, high throughput screenings (HTS), or the readout methods used (FACS, plate reader, microscope, etc.). For instance, fluorescent reporters represent a better choice for observing intracellular localization in cells or for ex vivo imaging of animal organs, because fluorescent signals in vivo are not powerful enough and the background in animal tissues dampers detection. Conversely, for quantitative determinations during in vitro or in vivo studies, luciferase proteins represent a more convenient option [24,26,28,29,65,66,68,69,70,83,84,85]. Therefore, multiple reporter genes with different properties have been used for the development of replication-competent IAVs. Because of this necessity of selecting the best reporter for each study, we have also developed a replication-competent ΔNS1 virus expressing a small luciferase (ΔNS1 Nluc) (Figure 6), which will be useful when bioluminescence readouts are preferable. Importantly, these results demonstrate the flexibility of our approach and expand the potential applications of our system. For instance, expression of foreign sequences such as immunomodulators or viral antigens in the background of an attenuated and safe ΔNS1 virus backbone opens the opportunity for the development of novel IAV vaccines or the use of ΔNS1 IAV as a vaccine vector for the prevention of other viral infections [11,21,25].

## Data Availability

The data that support the findings of this study are available from the corresponding author upon reasonable request.
